# Too Good to be Liked? When and How Prosocial Others are Disliked

**DOI:** 10.3389/fpsyg.2021.701689

**Published:** 2021-08-19

**Authors:** Lucia L.-A. Boileau, David J. Grüning, Herbert Bless

**Affiliations:** ^1^Chair of Microsociology and Social Psychology, Department of Psychology, School of Social Sciences, University of Mannheim, Mannheim, Germany; ^2^Department of Cognitive Research in Social Psychology, Institute of Psychology, Faculty of Behavioral and Cultural Studies, Heidelberg University, Heidelberg, Germany

**Keywords:** devaluation, do-gooder derogation, social comparison, communal narcissism, prosocial behavior

## Abstract

Outstandingly prosocial individuals may not always be valued and admired, but sometimes depreciated and rejected. While prior research has mainly focused on devaluation of highly competent or successful individuals, comparable research in the domain of prosociality is scarce. The present research suggests two mechanisms why devaluation of extreme prosocial individuals may occur: they may (a) constitute very high comparison standards for observers, and may (b) be perceived as communal narcissists. Two experiments test these assumptions. We confronted participants with an extreme prosocial or an ordinary control target and manipulated comparative aspects of the situation (salient vs. non-salient comparison, Experiment 1), and narcissistic aspects of the target (showing off vs. being modest, Experiment 2). Consistent with our assumptions, the extreme prosocial target was liked less than the control target, and even more so when the comparison situation was salient (Experiment 1), and when the target showed off with her good deeds (Experiment 2). Implications that prosociality does not always breed more liking are discussed.

## Introduction

Imagine Lisa, who exhibits considerable prosocial behavior. In her free time, she helps disabled people and supports disadvantaged children. She is concerned with the environment and is actively engaged in respective behaviors. Considering all these positive behaviors of Lisa, and compared to other persons who do not exhibit as much prosocial behavior—would you like her? Chances are that many observers would. If you question, however, that you would like Lisa, then your reaction is in line with scientific research suggesting that responses to prosocial individuals and groups are not always positive. For example, Parks and Stone (2010) demonstrated that individuals who act particularly unselfish and prosocial in a standardized group setting were likely to be expelled from the group by other group members—who in fact benefitted from the prosocial behaviors. Though this observation is rather surprising, it converges with other findings that suggest depreciation and rejection of very prosocial targets (Fisher et al., 1982; Herrmann et al., 2008; Pleasant and Barclay, 2018).

With respect to the underlying mechanism for this dislike and rejection of very prosocial group members “it is very unclear why it occurs” (Parks et al., 2013, p. 143). In the present research, we suggest two possible mechanisms to address the question why outstandingly prosocial others are sometimes disliked by others.

First, in line with research on social comparison (Alicke, 2000; Mussweiler, 2003; Bless and Schwarz, 2010; Morina, 2021) one may speculate that a very prosocial person constitutes a very high standard of comparison and that such standards have direct (negative) consequences for the evaluation of one's own behaviors (Parks and Stone, 2010). Consequently, one might dislike the comparison target (i.e., the outstandingly prosocial target).

Second, in line with research on communal narcissism (Gebauer et al., 2012; Nehrlich et al., 2019) one may speculate that a very prosocial person is perceived as someone who exerts “excessive self-enhancement” in the domain of prosociality and who is showing off with her good deeds (Rentzsch and Gebauer, 2019; p. 1373). Consequently, one might dislike the presumable communal narcissist (i.e., the outstandingly prosocial target).

We readily admit that other mechanisms may account for the derogation of prosocial targets. In light of the limited evidence on the causes of dislike of prosocial targets (see Parks et al., 2013), we focus on these two aspects as they reflect two complementary sources of potential dislike: (a) the role of the perceiver (i.e., dislike results from the social comparison component) and (b) the role of the target (i.e., dislike results from the (perceived) motives of the prosocial target; for beliefs about the motives see, Carlson and Zaki, 2021). Before outlining our research, we elaborate on both postulated mechanisms, that is, social comparison and perception of communal narcissism.

### Social Comparison as a Source of Disliking Prosocial Targets

A general principle of evaluative judgment suggests that by applying a higher comparison standard the judgmental target will be evaluated less positively (Bless and Schwarz, 2010). As individuals' self-evaluation is considerably influenced by social comparisons (Morina, 2021), one may conclude that being confronted with a very prosocial other makes observers “uncomfortable” (Parks et al., 2013, p. 143). Consequently, observers may try to avoid such high comparison standards, for example by expelling outstanding prosocial others from their group (Parks and Stone, 2010). If such an exclusion from the group is not possible or too costly, other mechanisms can be applied to separate oneself from the comparison standard. One possibility in this respect may be reflected in a dislike of the prosocial person which increases the social distance between the observer and the comparison standard ^1^.

In this regard, prior research provides many examples of negative responses toward those who are more attractive, who perform better, or who possess more: When confronted with superior others, individuals tend to grudge them their success and deny them their attractiveness, feel resentment, and envy, express gloating if they fail, show unwillingness to befriend and interact with them, and dislike them (e.g., Krebs and Adinolfi, 1975; Crosby, 1976; Bernstein and Crosby, 1980; Bers and Rodin, 1984; Salovey and Rodin, 1984; Olson and Hazlewood, 1986; Smith et al., 1996; Feather and Sherman, 2002; Agthe et al., 2011; Crusius and Mussweiler, 2012; Kim et al., 2018; Hartwich and Becker, 2019; Crusius et al., 2020).

Interestingly, empirical research on negative responses toward superior comparison targets has mostly focused on the performance domain, including different facets such as intellectual, academic, or financial performance. Although some studies suggest that prosocial individuals sometimes experience bullying, dislike, and low respect from others as well (Fisher et al., 1982; Herrmann et al., 2008; Parks and Stone, 2010; Pleasant and Barclay, 2018), empirical evidence on the comparison aspect is rare in the domain of prosocial behavior. In research addressing performance and ability, comparison processes are, for example, tackled by manipulating participants' involvement (Alicke et al., 1997; Lassiter and Munhall, 2001), or testing self-relevant against self-irrelevant comparison dimensions (Pleban and Tesser, 1981; Salovey and Rodin, 1984).

One study so far provides some indirect support for the social comparison perspective in the prosociality domain (Parks and Stone, 2010). Participants were asked in an open answer format to describe why they had rejected co-players who played very cooperatively (vs. selfishly) in a public goods game. The authors found that some participants rejected the cooperator because they considered them as norm-dissenters, but others rejected them because they felt inferior in comparison. That is, social comparison processes may have at least partly played a role in the devaluation of the cooperative other.

### Perceived Communal Narcissism as a Source of Disliking Prosocial Targets

While outstandingly prosocial individuals might be devaluated because they are potentially perceived as very high comparison standards, devaluation also occurs for other reasons. Such other sources for a dislike of prosocial targets may rest in the (perceived) motives attributed to the prosocial behavior. In this respect, researchers have argued that the basic motives to behave prosocially can be either altruistic or egoistic in nature (Batson and Coke, 1981; Dovidio, 1984). On the one hand, prosocial behavior out of altruistic motives requires empathic feelings toward the respective other (Batson, 2017). When altruistic motives are at play, “benefits to self are not the ultimate goal of helping; they are unintended consequences” (Batson and Shaw, 1991, p. 114). On the other hand, individuals may act prosocially because of social norms (e.g., reciprocity; Gouldner, 1960; Cialdini et al., 1981), to maintain or restore positive self-views (Brown and Smart, 1991), or because they expect rewards (e.g., positive feelings or social recognition; Winterich et al., 2013; Aknin et al., 2018) ^2^.

The different motives for prosocial behavior are, at least in parts, reflected in perceivers' beliefs about motives of others, which in turn affects evaluative judgements of prosocial others (Carlson and Zaki, 2021). Dependent on the perceived motives, perceivers may, for example, conclude that outstandingly prosocial individuals are seeking attention from others for their good deeds. In turn, outstandingly prosocial individuals may be accused of being arrogant, and less liked. Indeed, research in the domain of morally motivated behavior (i.e., vegetarianism) suggests that moral individuals may be accused of being show-offs, who are “arrogant,” “conceited,” “pretentious,” or “posers” (Minson and Monin, 2012, p. 202).

Such show-off tendencies in the domain of prosociality are captured in the concept of communal narcissism (Gebauer et al., 2012; Nehrlich et al., 2019). Specifically, communal narcissism is defined as excessive self-enhancement in the domain of communion^3^ (e.g., “I am the most helpful person I know,” “I'll make the world a much more beautiful place;” Communal Narcissism Inventory; Gebauer et al., 2012). Self-reported communal narcissism has been linked to higher arrogance and self-esteem (Gebauer et al., 2012; Zemojtel-Piotrowska et al., 2017). Consequently, communal narcissists might be perceived as arrogant show-offs by others as well, and thus, as less likable (Rentzsch and Gebauer, 2019).

Indeed, self-presentation strategies have been associated with reduced liking (Vonk, 1999). Past research on narcissism similarly suggests that narcissists are liked less than their non-narcissistic counterparts (Czarna et al., 2014, 2016; Leckelt et al., 2015; Back et al., 2018; Rentzsch and Gebauer, 2019). However, most of this research has only focused on agentic narcissism, which is distinct from communal narcissism (Gebauer et al., 2012) and pertains to excessive self-enhancement in the domain of agency (e.g., competence, performance). Thus, just as for social comparison research, research on narcissism is surprisingly scarce when it comes to investigating and explaining the devaluation of perceived prosocial (i.e., communal) others—whereas research on the devaluation of perceived competent (i.e., agentic) others is much more advanced.

One study found some evidence for a negative evaluation of communal narcissists: adolescents ascribed higher aggressiveness to peers who scored high on communal narcissism (Barry et al., 2017). Moreover, another study that focuses on likability speculates about an “annoying show-off mentality” (Rentzsch and Gebauer, 2019, p. 1371), yet provides evidence for the opposite effect that communal narcissists could be liked more than non-narcissists. This pattern is attributed to others anticipating that communal narcissists would like them more, and in turn, therefore reporting higher liking of communal narcissists (tit-for-tat hypothesis, Rentzsch and Gebauer, 2019). It is thus far from obvious that communal narcissists would be liked less than their non-narcissistic counterparts in general.

Importantly, most studies focused on how self-reported narcissists are perceived by others, whereas research on other-reports of communal narcissism is scarce. To investigate other-reports of communal narcissism is particularly important in the present research. Self-reported communal narcissists do not actually show more prosocial behavior than their non-narcissistic counterparts but only report to do so (Nehrlich et al., 2019). That is, a discrepancy between self-reported, and objective or other-reported prosociality of communal narcissists exists (Barry et al., 2017; Nehrlich et al., 2019). It remains therefore unclear whether outstandingly prosocial individuals would actually self-report higher communal narcissism, or if they would only be perceived as higher in communal narcissism by others. Thus, when investigating the devaluation of very prosocial individuals, ascribed communal narcissism might play a larger role than self-reported communal narcissism.

### The Present Research

Building on past research in the domain of devaluation of others, we investigate the possibility that outstandingly prosocial individuals might be liked less than moderately prosocial individuals. Considering past research in the domains of social comparison and narcissism, we investigate the possibility that the reduced liking of outstandingly prosocial individuals might be due to perceiving them as (a) high comparison standards, and (b) as narcissists.

So far, research has investigated these two potential mechanisms mostly in the domain of competence (i.e., agency) rather than in the domain of prosociality (i.e., communion). That is, reduced liking has mostly been investigated (a) in situations where the comparison target was highly competent, rather than highly prosocial, and (b) with targets that were perceived as agentic narcissists, rather than communal narcissists. For various reasons, it is unclear whether and how existing research in the domain of agency, or competence, can be directly applied to the domain of communion, or prosociality (see Fiske et al., 2007; Gebauer et al., 2014).

First, being and appearing prosocial looms large for most people (Aquino and Reed, 2002), and can be even more self-relevant than being competent (Ybarra et al., 2012). The potentially high self-relevance may in turn influence how a target person is evaluated (Alicke, 2000). Second, acting prosocially tends to be more ambiguous than acting competent (Alicke, 2000). Whereas competence on many occasions is quantifiable, for example, through test scores or work status, prosociality is often less objective. Ambiguity might in turn facilitate biased target evaluations (Alicke, 2000). Third, competence and prosociality may differ in terms of (perceived) controllability. While it is very difficult to improve one's competence, improving one's prosociality may be easier to accomplish (Reeder and Brewer, 1979). In line with this notion, more personal responsibility may be attributed to deficits in prosociality vs. competence (cf. Monin et al., 2008; Minson and Monin, 2012; O'Connor and Monin, 2016). In combination, due to differences in self-relevance, ambiguity, and personal responsibility, a direct transfer of the findings from competence to the prosociality domain requires a closer look.

The present research aims at addressing this issue in two studies. Specifically, we investigate the assumption that targets can be liked less when they display unambiguously extreme prosocial behavior. To investigate the role of social comparison processes, we experimentally manipulate whether comparison with the prosocial target is made salient or not (Experiment 1). To investigate the role of perceptions of communal narcissism, we experimentally manipulate whether the target uses her prosocial behavior to show-off or not (Experiment 2).

Importantly, by focusing on a potential reduced liking we readily acknowledge that the devaluation of prosocial targets might also take on other forms (cf. Monin, 2007). In this respect, individuals may accuse the target of not being prosocial at all but having immoral motives instead (e.g., “She might be a helpful person, but she only wants credit for it”). This fits with literature on attribution, suggesting that people are prone to ascribe negative behavior of others to their personality, and positive behavior to external causes (Pettigrew, 1979; Ybarra, 2002). Consequently, the target would no longer be perceived as highly prosocial or moral.

Moreover, observers can shift their focus from the prosocial dimension to other dimensions of social judgment—as to the competence dimension (e.g., “She might be a helpful person, but she isn't smart”). Accordingly, prior research has demonstrated that altruistic co-players are sometimes evaluated as weak (Liebrand et al., 1986). As we wanted to focus on the reduced liking of the target, we created situations in which the target behaved undoubtedly prosocial.

## Experiment 1

Experiment 1 was designed to investigate the possibility that a target person is liked less when she displays outstandingly prosocial behavior, and that the reduced liking is due to the target constituting a high comparison standard. We therefore confronted a random half of the participants with a target person who displayed numerous unequivocally prosocial activities. The other random half was confronted with a control target who was described positively but did not exhibit extraordinarily prosocial activities. We further manipulated participants' attention toward the comparison. Therefore, half of the participants were informed that they would first evaluate the target and then themselves on the very same items. The other half did not receive this information. We assumed that informing participants about the subsequent self-rating triggers comparison processes.

Based on previous considerations, we hypothesized the prosocial target to be liked less than the control target. Moreover, we expected this effect to be more pronounced when the comparison situation is salient than when it is not salient.

Because we aimed at eliminating other possibilities of target devaluation besides reduced liking (e.g., questioning the targets' prosociality or competence) we provided information on activities that were unquestionably prosocial, and further presented the target as capable of managing these different challenging and time-consuming prosocial activities. To test the success of this approach, we assessed a set of items related to the prosociality dimension and expected the prosocial target to be perceived as more prosocial than the control target. Moreover, a set of items related to the competence dimensions was assessed to investigate possible devaluations on perceived competence. Given that we attempted to exclude ambiguous interpretations of the target's actual behavior itself, we expected these effects to be independent of the salience of the comparison.

To obtain some preliminary information about the role of our second mechanism, perceived communal narcissism in the target, we further included a set of items to assess perceived communal narcissism (Gebauer et al., 2012).

Finally, we were interested in what participants thought about how the target sees herself (e.g., whether she sees herself as highly prosocial). For explorative reasons, participants were therefore asked about their opinion on how the target sees herself on the same set of items. Afterwards, participants evaluated themselves on the same items as they evaluated the target, to keep up with our cover story. Again, no specific hypotheses were formulated. Finally, for explorative reasons, we assessed participants' self-esteem, their self-reported communion and agency, and self-monitoring. In the following, we report all variables, conditions, and exclusions of Experiment 1.

### Methods

#### Participants and Design

A total of 253 participants were recruited *via* an online-recruitment platform of our university. This sample size was not increased after analysis. Participation was rewarded either with course credit or with the chance to win one of four vouchers to the value of 25€. The sample consisted primarily of students (96.8%) with 208 (82.2%) female and 45 (17.8%) male participants. Age ranged from 18 to 49 years (*M* = 23.11, *SD* = 4.99). Participants were randomly assigned to the conditions of a 2 (target type: extreme prosocial vs. control) x 2 (comparison: salient vs. not salient) factorial design.

#### Procedure

Participants were presented with an ostensible newspaper article that featured a fictitious student named “Lisa.” By choosing a “student” target, we increased the potential relevance for most of our participants (i.e., students). Participants learned about Lisa's life situation, hobbies, and social activities. Subsequently, they filled in the questionnaire in the following order: (1) questions about how Lisa perceives herself on communal narcissism, likeability, competence, and prosociality; (2) questions about how they perceive Lisa on communal narcissism, likeability, competence, and prosociality; and (3) questions about how they perceive themselves on communal narcissism, likeability, competence, prosociality, self-esteem, agency, communion, self-monitoring, and similarity to the target. Finally, participants were thoroughly informed about the scientific purpose of the experiment. The complete questionnaire with the original wording (German) can be found on https://osf.io/2dtps/?view_only=d43d64133da54d4f9fefa461e198f956.

#### Measures

*Independent Variables*. The type of target was manipulated by varying the content of the newspaper article. In the extreme prosocial condition, Lisa was portrayed as an extraordinarily prosocial student. Specifically, she was described as someone dedicated to help disabled people and disadvantaged children, concerned about the environment, politically active, and a very loyal friend. In the control condition, Lisa was described as an ordinary student. Specifically, we provided a somewhat positive but in general neutral description of her living conditions, side jobs, and travel plans.

To manipulate comparison salience, participants received different instructions before reading the newspaper article. In the comparison salient condition, participants were informed that, after reading the newspaper article, they would first evaluate the described person and then they would rate themselves on the same dimensions. In the not salient condition participants were informed that they would evaluate the target person, but it was not mentioned that they would have to rate themselves.

*Dependent Variables and Control Variables*. After participants had read the newspaper article, we assessed likeability as the central dependent variable with three items (e.g., “Lisa is a likable person“) on a scale ranging from 1 to 9 with higher values indicating more liking (α = 0.95).

Subsequently, to allow for testing whether Lisa was unambiguously perceived as prosocial and competent, we assessed perceived prosociality and competence with six and three self-conceptualized items, respectively (e.g., “Lisa behaves ethically correct” and “Lisa works effectively,” resp.). Higher values on a scale ranging from 1 to 9 indicate higher prosociality (α = 0.87), and higher competence (α = 0.88), respectively.

We further assessed how participants perceived the targets with respect to communal narcissism (Gebauer et al., 2012) with three adapted items (e.g., “Lisa wants others to see that she makes the world a better place,” α = 0.87). We also included some other measures for explorative reasons: We asked participants to take Lisa's perspective and to indicate how Lisa perceived herself (i.e., assumed target self-evaluations) regarding her likeability (α = 0.91), prosociality (α = 0.84), and competence (α = 0.87), and whether she perceives herself in a narcissistic way (α = 0.84). Participants were presented with the same items described above. For example, participants indicated their beliefs regarding whether Lisa herself thinks “I am a likable person.” In order to keep up with the cover story, participants then evaluated themselves (i.e., rater self-evaluations) with regard to their own likeability (α = 0.94), prosociality (α = 0.70), and competence (α = 0.78) on the same items and filled in the measure on communal narcissism (α = 0.65). Finally, participants worked on short versions of questionnaires pertaining to agency (α = 0.47) and communion (α = 0.51), respectively (four items each, adapted from Gebauer (2021), self-esteem (four items, α = 0.87, Von Collani and Herzberg, 2003), and self-monitoring (eight items, α = 0.68, Graf, 2004), respectively, and perceived similarity between the rater and the target (one item).

At the end of the questionnaire, participants were asked about their gender, age, profession, and native language.

### Results

#### Liking

Participants' ratings of how much they liked Lisa were entered into a two-way ANOVA. As can be seen in Table 1, the prosocial target was liked less than the control target, *F*_(1, 249)_ = 14.92, *p* < 0.001, η^2^_p_ = 0.01. Importantly, and in line with our hypothesis, the devaluation of the target was more pronounced when the comparison was made salient, *t*(249) = −4.19, *p* < 0.001, than when the comparison was not made salient, *t*(249) = −1.31, *p* = 0.19, which is reflected in a significant interaction of target type and comparison salience, *F*_(1, 249)_ = 4.31, *p* = 0.04, η^2^_p_ = 0.02. No main effect of comparison salience was obtained, *F* < 1. A sensitivity power analyses revealed that with the given sample size (*N* = 253), an assumed alpha level of 0.05 and a power criterion of 0.80, Experiment 1 would have allowed us to observe effect sizes of η^2^ = 0.03 for the main effect of target type and the interaction effect, respectively, which represents small to medium effect sizes.

**Table 1:** Mean target likeability ratings per condition in Experiment 1.

	**Control target**		**Prosocial target**	
	***M***	***SD***	***M***	***SD***
Comparison not salient	6.79	1.83	6.41	1.65
Comparison salient	7.20	1.30	5.96	1.80

#### Prosociality and Competence^4^

To test whether the prosocial target was unequivocally described as prosocial and competent, participants' ratings of Lisa's prosociality and competence were entered into two-way ANOVAs. As displayed in Table 2 the prosocial target was perceived as more prosocial, *F*_(1, 249)_ = 158.28, *p* < 0.001, η^2^_p_ = 0.29, and more competent, *F*_(1, 249)_ = 31.26, *p* < 0.001, η^2^_p_ = 0.08, than the control target. Importantly, prosociality and competence were independent of comparison salience, reflected in non-significant main effects, *F* < 1, and interaction effects with target type, *F*_(1, 249)_ = 3.00, *p* = 0.08, η^2^_p_ = 0.01, and *p* > 0.25, respectively^5^. This pattern suggests the description was unambiguous in this respect and that participants did not question the prosocial target's prosociality and competence; neither overall nor as a response to comparison salience.

**Table 2:** Mean target prosociality and competence ratings per condition in Experiment 1.

	**Control target**		**Prosocial target**	
	***M***	***SD***	***M***	***SD***
Prosociality
Comparison not salient	5.91	1.23	7.90	0.85
Comparison salient	6.09	1.17	7.60	1.16
Competence
Comparison not salient	6.60	1.56	7.77	1.20
Comparison salient	6.90	1.51	7.69	1.31

#### Exploratory Analyses

*Communal Narcissism*. A two-way ANOVA revealed that participants attributed more communal narcissism to the prosocial target than to the control target, *F*_(1, 249)_ = 95.30, *p* < 0.001, η^2^_p_ = 0.18 (view Table 3). Thus, although our prosocial target was positively perceived regarding prosociality and competence, she was perceived more negatively when it came to communal narcissism. Importantly, no main or interaction effect involving comparison salience emerged, *F* < 1, suggesting that perceived communal narcissism in the prosocial target may be independent of perceiving the target as high comparison standard.

**Table 3:** Mean target communal narcissism ratings per condition in Experiment 1.

	**Control target**		**Prosocial target**	
	***M***	***SD***	***M***	***SD***
Comparison not salient	5.20	1.49	7.33	1.72
Comparison salient	5.51	1.56	7.38	1.76

*Assumed Target Self-Evaluations*. We further ran two-way ANOVAs to test whether participants differed in how they assumed Lisa would perceive herself. Participants expected the prosocial target to perceive herself as more prosocial, *F*_(1, 249)_ = 166.29, *p* < 0.001, η^2^_p_ = 0.27, as more competent, *F*_(1, 249)_ = 43.90, *p* < 0.001, η^2^_p_ = 0.11, to think about herself in a more communal narcissistic way, *F*_(1, 249)_ = 135.98, *p* < 0.001, η^2^_p_ = 0.24, but not to perceive herself as more likable than the control target, *F* < 1. Assumed target self-evaluation ratings were independent of comparison salience (main effects: *p* > 0.20 for communal narcissism, *F* < 1 for all other variables; interaction effects with target type: *p* > 0.15 for competence, *F* < 1 for all other variables).

*Perceived Similarity*. We further tested whether participants differed in how similar they perceived themselves and the target. Participants indicated that they were more similar to the control target than to the prosocial target, *F*_(1, 249)_ = 15.92, *p* < 0.001. No significant main or interaction effects involving comparison salience emerged, *F* < 1, and *F*_(1, 249)_ = 3.15, *p* = 0.08, η^2^_p_ = 0.01^6^.

*Self-Evaluations*. Participants in the prosocial condition rated themselves as marginally more likable, *F*_(1, 247)_ = 3.09, *p* = 0.08, η^2^_p_ = 0.003, and less prosocial, *F*_(1, 247)_ = 2.97, *p* = 0.09, η^2^_p_ = 0.005, than participants in the control condition. Participants in the salient condition indicated marginally higher self-esteem than participants in the non-salient condition, *F*_(1, 248)_ = 3.33, *p* = 0.07, η^2^_p_ = 0.01. All other main effects of target type and comparison salience were not significant (target type on agency: *p* > 0.20; on all others: *F* < 1; comparison salience on likeability and agency: *p* > 0.15; on all others: *F* < 1). All interaction effects were not significant (on prosociality: *p* > 0.25; all others: *F* < 1).

### Discussion

The results obtained in Experiment 1 demonstrate two important aspects. First, they indicate that a target performing numerous prosocial behaviors in an outstanding way may nevertheless be devaluated. This devaluation is reflected in a reduced liking of the target person. Such a devaluation is in line with prior research demonstrating that exemplary prosocial behavior can sometimes receive depreciation and rejection from others (e.g., Fisher et al., 1982; Herrmann et al., 2008; Parks and Stone, 2010; Pleasant and Barclay, 2018).

Second, the present research suggests that comparison processes play an important role in the mechanisms that underlie the devaluation. Specifically, consistent with our hypotheses, the devaluation was most pronounced when the experimental situation made comparisons between the prosocial target and the observer particularly likely. Although prior research has alluded to potential comparison processes (Parks and Stone, 2010; Parks et al., 2013), a direct test of the causal role of comparison processes in the prosocial domain has so far not been done. The present research, thus, provides first direct evidence for the causal role of comparison processes on the devaluation of extreme prosocial targets. We address other implications of the obtained findings in the General Discussion.

## Experiment 2

The reported findings of Experiment 1 demonstrate that prosocial targets can be devaluated by reduced liking even though both their competence and their prosociality are evaluated very positively. Moreover, the results point to comparison processes as a crucial factor involved in the devaluation. However, they also point to the possibility that perceived communal narcissism in the prosocial target might play a role above and beyond comparison processes.

Experiment 2 serves two main purposes. First, Experiment 2 was designed as a conceptual replication of the main effect found in Experiment 1 (i.e., the prosocial target being liked less than a control target). Second, Experiment 2 introduces a novel mechanism that might explain the reduced liking of the prosocial target above and beyond social comparison processes: perceived communal narcissism in the target.

With Experiment 2, we shift from features of the situation to characteristics of the target. Specifically, we varied the degree to which communal narcissism could be attributed to the target. As one central aspect of communal narcissism is that others ought to see one's own contributions (e.g., “I will be well known for the good deeds I will have done,” Communal Narcissism Inventory: Gebauer et al., 2012), we manipulated whether or not the target was interested in making her prosocial behavior public (show-off condition) or whether the target tried to remain anonymous (modest condition) with respect to her prosocial behavior. We hypothesized that the prosocial target is devaluated relative to the control target but that this devaluation is more pronounced when attribution of communal narcissism to the target is more likely (i.e., in the show-off rather than the modest condition). These hypotheses were pre-registered on *AsPredicted*^7^ In the following, we report all variables, conditions, and exclusions of Experiment 2.

### Methods

#### Participants and Design

A total of 203 participants were recruited *via* a university online-recruitment platform. Participants were randomly assigned to the conditions of a 2 (target type: extreme prosocial vs. control) x 2 (show-off vs. modest) factorial design. This sample size was not increased after analysis. Participation was rewarded either with course credit or with the chance to win one of four vouchers to the value of 25€ 0.56 participants had to be excluded after incorrectly answering an attention check question with respect to the show-off/modest manipulation (see below). The final sample consisted of 147 participants (90.5% female), with ages ranging from 18 to 42 years (*M* = 21.37, *SD* = 3.45). All participants except one were students.

#### Procedure

The procedure was identical to that in Experiment 1 except that participants received different instructions before reading the ostensible newspaper article about Lisa (see below), and that we did not include assumed target self-evaluations. Participants filled in the questionnaire in the following order: (1) questions about how they perceive Lisa on communal narcissism, modesty, likeability, competence, and prosociality; and (2) questions about how they perceive themselves on communal narcissism, modesty, likeability, competence, prosociality, self-esteem, and similarity to the target.

#### Measures

*Independent Variables*. The type of target was manipulated exactly as in Experiment 1, that is, by varying the content of the newspaper article. In the extreme prosocial condition, Lisa was portrayed as an extraordinarily prosocial student. In the control condition, Lisa was described as an ordinary student.

To manipulate the degree to which communal narcissism could be attributed to the target, participants received different additional information about Lisa before reading the newspaper article. First, all participants were given the information that the article was written for the “World Student Day” and that portrayed students could either come forward themselves for an interview or be suggested by family and friends. In the show-off condition, participants were informed that Lisa came forward herself, had already given many interviews, and that she had no problem with being mentioned by her real name in the article. In the modest condition, participants were informed that Lisa was suggested by her friends, had never given an interview before, and that Lisa is not her real name as she wanted to stay anonymous.

*Dependent Variables and Control Variables*. After participants had read the newspaper article, we used the same items as in Experiment 1 to assess target likeability as the central dependent variable (α = 0.97). Participants rated target prosociality (α = 0.81) and competence (α = 0.78) to allow for testing whether Lisa was unambiguously perceived as prosocial and competent. Perceived communal narcissism (α = 0.85) was assessed to test whether the show-off/modest manipulation was successful. In addition to Experiment 1, we also included four items on perceived modesty (e.g., “Lisa thinks that she is an average person,” α = 0.61, Rammstedt et al., 2012; Schreiber and Iller, 2016; Schreiber et al., 2018) as a positively connoted counterpart to communal narcissism.

We further asked participants to evaluate themselves (i.e., rater self-evaluations) regarding likeability (α = 0.88), prosociality (α = 0.67), competence (α = 0.73), communal narcissism (α = 0.73) and modesty (α = 0.63). Finally, participants worked on the same self-esteem measure (α = 0.92) and answered the same question about perceived target-rater similarity as in Experiment 1. Unlike in Experiment 1, we did not measure participants' self-evaluations on self-monitoring, agency and communion.

Finally, participants worked on two questions that checked for whether they had understood the show-off/modest manipulation (e.g., “Did Lisa herself or her friends suggest Lisa as a target for the newspaper article?”). Participants were then asked about their gender, age, profession, and native language.

### Results

#### Liking

Participants' ratings of how much they liked Lisa were entered into a two-way ANOVA. As can be seen in Table 4, the prosocial target was again liked less than the control target, *F*_(1, 143)_ = 17.33, *p* < 0.001, η^2^_p_ = 0.02. Importantly, and in line with our hypothesis, the devaluation of the target was more pronounced when the target actively went public, *t*(143) = −4.26, *p* < 0.001, than when she preferred anonymity, *t*(143) = −1.72, *p* = 0.09, reflected in a marginally significant interaction, *F*_(1, 143)_ = 3.25, *p* = 0.07, η^2^_p_ = 0.02. A sensitivity power analyses revealed that with the given sample size (*N* = 147), an assumed alpha level of 0.05 and a power criterion of 0.80, Experiment 2 would have allowed us to observe effect sizes of η^2^ = 0.05 for the main effect of target type and the interaction effect, respectively, which represents medium effect sizes.

**Table 4:** Mean target likeability ratings per condition in Experiment 2.

	**Control target**		**Prosocial target**	
	***M***	***SD***	***M***	***SD***
Modest	6.96	1.81	6.24	1.81
Show-Off	6.98	1.42	5.19	2.11

#### Prosociality and Competence

To test whether the prosocial target was unequivocally described as prosocial and competent, participants' ratings of Lisa's prosociality and competence were entered into two-way ANOVAs. As displayed in Table 5 the prosocial target was perceived as more prosocial, *F*_(1, 143)_ = 86.16, *p* < 0.001, η^2^_p_ = 0.28, and more competent, *F*_(1, 143)_ = 8.57, *p* = 0.004, η^2^_p_ = 0.03, than the control target. Moreover, the modest target was perceived as more prosocial than the show-off target, *F*_(1, 143)_ = 4.28, *p* = 0.04, η^2^_p_ = 0.002. All other main effects and interactions were not significant, *p* > 0.20. This pattern suggests that the description of the prosocial target was unambiguous in this respect and that participants did not question the prosocial target's prosociality and competence; neither overall nor as a response to the show-off/modest information.

**Table 5:** Mean target prosociality and competence ratings per condition in Experiment 2.

	**Control target**		**Prosocial target**	
	***M***	***SD***	***M***	***SD***
Prosociality
Modest	6.10	1.21	7.86	0.83
Show-Off	5.98	0.98	7.31	1.03
Competence
Modest	7.11	1.36	7.75	0.90
Show-Off	6.93	1.34	7.48	1.36

#### Communal Narcissism

A two-way ANOVA revealed that participants attributed more communal narcissism to the show-off target than to the modest target, *F*_(1, 143)_ = 13.58, *p* < 0.001, η^2^_p_ = 0.001 (see Table 6), indicating the success of the show-off/modest manipulation. Moreover, participants perceived more communal narcissism in the prosocial target than in the control target, *F*_(1, 143)_ = 82.55, *p* < 0.001, η^2^_p_ = 0.12. Thus, the prosocial target was perceived more negatively when it came to communal narcissism. This effect was more pronounced in the show-off condition, reflected in a significant interaction effect, *F*_(1, 143)_ = 9.23, *p* = 0.003, η^2^_p_ = 0.06.

**Table 6:** Mean target communal narcissism and modesty ratings per condition in Experiment 2.

	**Control target**		**Prosocial target**	
	***M***	***SD***	***M***	***SD***
Communal narcissism
Modest	4.23	1.55	5.91	1.94
Show-Off	4.39	1.57	7.71	1.44
Modesty
Modest	5.04	1.29	3.75	1.23
Show-Off	3.72	1.13	3.12	1.11

#### Modesty

A two-way ANOVA revealed that participants attributed less modesty to the show-off target than to the modest target, *F*_(1, 143)_ = 24.20, *p* < 0.001, η^2^_p_ = 0.13 (see Table 6), indicating the success of the show-off/modest manipulation. Moreover, participants perceived less modesty in the prosocial target than in the control target, *F*_(1, 143)_ = 21.73, *p* < 0.001, η^2^_p_ = 0.13. Thus, the prosocial target was perceived more negatively when it came to modesty. Importantly, this effect was less pronounced in the show-off condition, reflected in a marginally significant interaction effect, *F*_(1, 143)_ = 3.05, *p* = 0.08, η^2^_p_ = 0.02. Thus, the obtained pattern of means suggests that modesty and communal narcissism might capture not exactly opposed, but rather different aspects. We return to this issue in the General Discussion section.

#### Mediation Analysis

We tested whether our central interaction effect of target type by show-off/modest on target likeability was mediated *via* perceived target communal narcissism (bivariate Pearson correlations between target communal narcissism and likeability: *r* = −0.44, *p* < 0.001). To do so, we ran a structural equation model (mediated moderation) with the lavaan package in R (Rosseel, 2012) to test indirect and direct effects on likeability. We did not test a mediated moderation with perceived target modesty, as the crucial interaction effect of target type by show-off/modest on modesty did not point in the predicted, but in the opposite direction in the first place.

**Figure 1:**
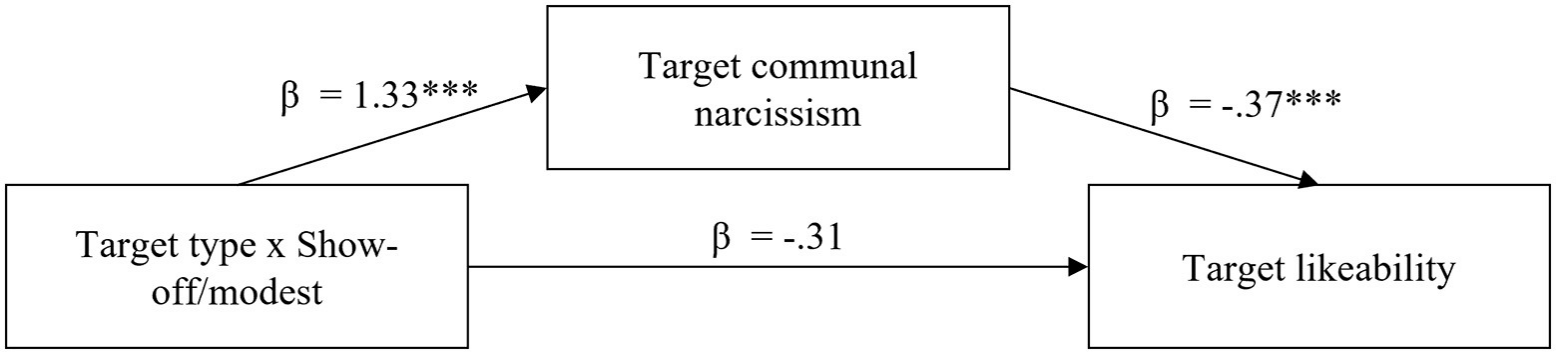
Moderated Mediation Model in Experiment 2. ***p < 0.001, **p < 0.01, *p < 0.05.

Results of the moderated mediation model are displayed in Figure 1. The indirect effect of the target type by show-off/modest interaction *via* communal narcissism on likeability was significant (β = −0.49, *p* < 0.001), whereas the direct effect of the interaction was not significant (β = −0.31, *p* = 0.19). That is, communal narcissism appears to fully explain the greater reduced liking of the prosocial target compared to the control target in the show-off compared to the modest condition. Importantly, we acknowledge that problems of causality (i.e., whether communal narcissism judgements caused reduced liking, or *vice versa*) and the possibility of other underlying mechanisms (which we might not have surveyed at all) cannot be ruled out by applying mediation models (Spencer et al., 2005; Fiedler et al., 2018).

#### Exploratory Analyses

*Perceived Similarity*. As in Experiment 1, participants indicated that they were more similar to the control target than to the prosocial target, *F*_(1, 143)_ = 10.64, *p* = 0.001, η^2^_p_ = 0.05, all main and interaction effects involving the manipulation of communal narcissism (i.e., show-off vs. modest) were not significant, *F* < 1.

*Self-Evaluations*. Participants in the prosocial condition rated themselves as less prosocial, *F*_(1, 143)_ = 4.04, *p* = 0.05, η^2^_p_ = 0.03, and more modest, *F*_(1, 143)_ = 6.99, *p* = 0.01, η^2^_p_ = 0.02, than participants in the control condition. Participants in the show-off condition reported marginally higher self-esteem than participants in the modest condition, *F*_(1, 143)_ = 3.53, *p* = 0.06, η^2^_p_ = 0.001. All other main effects of target type and show-off/modest were not significant, *F* < 1. All interaction effects were not significant (on prosociality, communal narcissism, and self-esteem: *p* > 0.15; all others: *F* < 1).

### Discussion

As in Experiment 1, the findings of Experiment 2 reveal that the target performing numerous prosocial behaviors was liked less than the control target, although participants recognized the outstanding prosocial behavior. This finding is in line with prior research that has pointed out potential devaluations of prosocial targets (e.g., Fisher et al., 1982; Herrmann et al., 2008; Parks and Stone, 2010; Pleasant and Barclay, 2018).

The devaluation was particularly pronounced when the target actively sought out publicity for her prosocial behavior, that is, if attribution of communal narcissism was more likely. The observed effects were indeed mediated by the perceived degree of communal narcissism in the prosocial vs. the control target—thus pointing to the role of attributions of communal narcissism.

One might speculate that attributions of communal narcissism come along with ascribing immoral motives, and thus, low prosociality, to the target. Such a mechanism might as well explain the reduced liking effect we observed. However, the prosocial target was always perceived as more prosocial than the control target, independent of whether she showed her behavior publicly (show-off condition) or anonymously (modest condition). This points to the role of ascribing communal narcissism as an unappealing show-off characteristic to the target without denying her prosociality. We address other implications of the obtained findings in the General Discussion section.

## General Discussion

The present research demonstrates that individuals who perform an outstanding degree of prosocial behaviors may be devaluated—due to their prosocial behaviors. Specifically, across two experiments, the prosocial target was liked less than the control target. This consistent pattern is unlikely to be due to participants' perception that the displayed behaviors did not unambiguously reflect prosocial behavior: When explicitly evaluating prosociality, the prosocial target was clearly perceived as prosocial (and more so than the control target). The finding that prosocial behaviors may decrease rather than increase liking seems rather surprising at first glance. Past research suggests that liking and perceptions of prosociality in others are in fact very highly correlated (Imhoff and Koch, 2017). However, the observed devaluation is in line with prior empirical research suggesting that superior prosocial others are indeed sometimes devaluated through rejection and dislike (Fisher et al., 1982; Herrmann et al., 2008; Parks and Stone, 2010; Pleasant and Barclay, 2018).

The present research goes beyond prior research that has similarly demonstrated a possible disliking of prosocial targets by suggesting and investigating two possible underlying processes. Thus, it responds to the call that mediating mechanisms for the dislike of very prosocial targets are yet to be investigated (Parks et al., 2013).

First, the reduced liking of the prosocial target was more pronounced when comparisons between the target and the observers were induced by the information that observers would first evaluate the target and then themselves on the very same items. Eliciting such a comparison expectation increased disliking of the prosocial target. Presumably, in this situation, the extremely prosocial target constituted a very high comparison standard, and this high standard would have negative consequences for participants' evaluations of themselves (Mussweiler, 2003; Bless and Schwarz, 2010; Morina, 2021). This conclusion extends indirect evidence by Parks and Stone (2010) by providing an experimental manipulation of the assumed comparison component.

Second, as predicted, the dislike of the prosocial target was increased when perceptions of communal narcissism (Gebauer et al., 2012; Nehrlich et al., 2019) were elicited by informing participants that the target actively sought to let others know about her prosocial behaviors. This finding suggests that a target's prosocial behavior will not turn into more liking but backfire when that target is perceived as someone who exerts “excessive self-enhancement” in the domain of prosociality and who is showing off with her good deeds (Rentzsch and Gebauer, 2019; p. 1373).

Interestingly, the two proposed accounts (comparison processes, and communal narcissism) may in fact be related. The perception that an individual competes for higher status on the communal dimension (Gebauer et al., 2012) might elicit social comparison processes just as a situation in which comparison is made salient. Communal narcissists do show-off because they want to outperform others—which inevitably entails a comparison. In case the comparison is not only given on the side of the prosocial target but also on the side of the perceiver, one might speculate that we manipulated comparative aspects of the situation in Experiment 1 and comparative aspects of the target in Experiment 2. Such speculation may explain why assessed communal narcissism in Experiment 1 was strongly related to disliking the prosocial target. In turn, eliciting social comparison *via* the situation might increase perceptions of communal narcissism as a form of target devaluation (cf. Monin, 2007). Note, however, that we did *not* find a significant interaction effect of target type and comparison salience on perceptions of communal narcissism in Experiment 1.

In combination, the present research provides first evidence on two potential mechanisms to explain the devaluation of very prosocial others. The findings thus provide a first, yet important step, for investigating the processes that lead to the devaluation of prosocial targets.

### Open Issues and Caveats

Although the present research offers new insights into the potential devaluation of prosocial others, it is important to address several open issues and caveats. First, one may speculate about the degree of prosocial behavior that is necessary to elicit a dislike of the target (in combination with the comparison component). Of course, prosocial behavior does not necessarily lead to disliking. In this respect, it is important to point out that our target was very outstanding with respect to prosocial behavior. Thus, to elicit devaluation, the target might have to be perceived as “too good” or “too perfect” (note that self-devaluation tends to be elicited when the outstanding other is perceived as unreachable; Lockwood and Kunda, 1997). Evidence in this respect has been reported for the ability domain demonstrating that a superior target was liked more than an average person—if the superior target displayed some imperfections in other domains (Aronson et al., 1966). Further research is needed to address this issue systematically in the prosocial domain.

Second, our conclusion on the crucial role of comparison processes for the dislike of outstanding prosocial targets matches with research on the devaluation of targets that outperform others in the domain of performance and abilities (Pleban and Tesser, 1981; Salovey and Rodin, 1984; Alicke et al., 1997; Alicke, 2000; Lassiter and Munhall, 2001). The present findings thus suggest that similar processes may cause devaluation of both oustandingly capable, and outstandingly prosocial individuals (for a discussion of different, yet overlapping conceptualizations of ability vs. prosociality, see, e.g., Fiske et al., 2007; Gebauer et al., 2014; Abele et al., 2016). Similarly, these processes may also cause devaluation of outstandingly moral individuals (e.g., Monin et al., 2008; Minson and Monin, 2012). Importantly, despite some overlaps, prosociality and morality are not the same. Morality comprises being loyal, fair, law-abiding, and pure (Graham et al., 2013)—aspects that are at least partly independent of prosociality. However, given the similar patterns of dislike observed for superior targets in these various domains, it seems worthwhile to investigate communalities and differences between prosociality and morality in their underlying mechanisms.

Third, we did not find complementary patterns for perceived communal narcissism vs. perceived modesty in Experiment 2. The obtained findings do not allow for an answer to this issue. With respect to the concept of narcissism it might be interesting to investigate whether narcissism and modesty are located on different sides of the same dimension or whether the two concepts are at least partly unrelated to each other (for a discussion of the humility and grandiose narcissism dimension, see Miller et al., 2012; Gebauer and Sedikides, 2019). This relation might also depend on whether narcissism and modesty are measured *via* self-reports or other-reports. Interestingly, while self-rated communal narcissism has been conceptualized and investigated (Gebauer et al., 2012; Nehrlich et al., 2019), the perception of communal narcissism in others has so far received little systematic investigation (Rentzsch and Gebauer, 2019).

Fourth, the crucial interaction of target type and communal narcissism (i.e., show-off vs. modest condition) in Experiment 2 did not reach the conventional level of significance when tested two-sided. We readily acknowledge this aspect. Note, however, that we pre-registered our study so that one-sided tests statistics could potentially be applied. Due to the unexpected drop out (due to the attention check, see above) the conducted analyses were presumably underpowered, which constitutes a common problem in the field of psychological research (Maxwell, 2004).

Fifth, the reduced liking of the outstandingly prosocial target reflects a contrast effect. General models on context effects in social judgment (cf. Bless and Schwarz, 2010) hold that—under specified conditions—contrast effects may turn in assimilation effects. We readily subscribe to this possibility. One condition that might apply to the present research could rest in the perceived similarity between target and perceiver. In case perceivers assume a high overlap between themselves and the target, they may derive positive implications for themselves rather than devaluating the target (e.g., basking in the reflected glory, Cialdini and DeNicholas, 1989; see also Brown et al., 1992; for an overview on assimilation vs. contrast effects, see Bless and Schwarz, 2010).

Sixth, our sample predominantly consisted of females. As our target was female as well, this might have influenced our results (e.g., see Espinosa and Kovárík, 2015), for gender differences in prosocial behavior). To address this issue, further research needs to test potential gender differences in the evaluation of outstandingly prosocial others.

Finally, it needs to be pointed out that we minimized the potential ambiguity of the prosocial behavior. One could speculate that other devaluation mechanisms (i.e., denying prosociality, ascribing lower competence, etc.) might be at work when the prosocial behavior is more ambiguous and more open to interpretations.

### Outlook

While readily acknowledging the open issues addressed above, we strongly believe that the present research addresses important issues. We consistently demonstrated that performing prosocial behaviors may lead to reduced liking. Moreover, we showed that the reduced liking of prosocial individuals is triggered by both comparison processes and perceptions of communal narcissism. The present set of studies therefore show, to our knowledge, the first direct evidence on underlying mechanisms in the devaluation of very prosocial others and provide a basis for future research.

Leaving the laboratory situation, the obtained findings suggest that performing prosocial behaviors is no guarantee to be liked. In fact, in some instances, individuals may be disliked because of their prosocial behaviors. This observation is in line with the ambiguously connoted term “do-gooder,” which on the one hand describes the target's “desire and effort to help people” but on the other hand, points out to potential evaluations of the target's behavior as “wrong,” or “annoying” (see Merriam-Webster, n.d.). We assume that the ambivalence of the term “do-gooder” is rather widespread. If so, research needs to pay more attention to the devaluation of prosocial others, as it might constitute a potential obstacle to individuals' motivation for prosocial behavior.

## Data Availability Statement

The datasets presented in this study can be found in online repositories. The names of the repository/repositories and accession number(s) can be found below: Open Science Framework https://osf.io/2dtps/?view_only=d43d64133da54d4f9fefa461e198f956.

## Ethics Statement

Ethical review and approval was not required for the study on human participants in accordance with the local legislation and institutional requirements. The patients/participants provided their written informed consent to participate in this study.

## Author Contributions

LB, DG, and HB contributed to conception and design of the experiments reported in this work. DG organized the database. LB and DG performed the statistical analysis. LB wrote the first draft of the manuscript. LB and HB wrote sections of the manuscript. All authors contributed to manuscript revision, read, and approved the submitted version.

## Conflict of Interest

The authors declare that the research was conducted in the absence of any commercial or financial relationships that could be construed as a potential conflict of interest.

## Publisher's Note

All claims expressed in this article are solely those of the authors and do not necessarily represent those of their affiliated organizations, or those of the publisher, the editors and the reviewers. Any product that may be evaluated in this article, or claim that may be made by its manufacturer, is not guaranteed or endorsed by the publisher.

## Footnotes

^1^These considerations can also be linked to theoretical and empirical research on self-enhancement. Research suggests that comparisons can be driven by the motive for self-enhancement (Wood, 1989; Helgeson and Mickelson, 1995; Sedikides and Gregg, 2008; Hepper and Sedikides, 2012; Sedikides and Alicke, 2020).

^2^Moreover, other factors including personality traits, religiosity or gender have been demonstrated to influence prosocial behavior (Penner et al., 1995; Norenzayan and Shariff, 2008; Eagly, 2009).

^3^Note that “communion” is a central domain of social judgement which is sometimes also referred to as “warmth” and includes aspects such as morality and prosociality (Abele and Wojciszke, 2018, 2007; Fiske et al., 2007; Gebauer et al., 2014).

^4^Regarding our target evaluation measures, we proposed four groups of variables: target likeability, prosociality, competence, and communal narcissism. An exploratory factor analysis reproduced this pattern in essence and revealed three factors: likeability, prosociality/competence, and communal narcissism. As both target prosociality and competence were purposely high due to our target description, and thus, variances were low, they did not emerge as two separate factors. Results from the factor analysis can be found in the Supplementary Material.

^5^The interaction effect on prosociality was marginally significant with the prosocial target being evaluated as more prosocial than the control target when the comparison was salient (*M* = 7.60 vs. *M* = 6.09), and slightly more so when the comparison was not salient (*M* = 7.90 vs. *M* = 5.91).

^6^The interaction effect of target type and comparison salience was marginally significant with the prosocial target being perceived as less similar than the control target when the comparison was salient (*M* = 3.94 vs. *M* = 5.27), and slightly less so when the comparison was not salient (*M* = 4.23 vs. *M* = 4.75).

^7^ https://aspredicted.org/ca9c8.pdf
