# Supplementary material for: Too Good to be Liked? When and How Prosocial Others are Disliked
Source: Front Psychol. 2021 Aug 19;12:701689. doi: 10.3389/fpsyg.2021.701689 (PMC8417242; doi:10.3389/fpsyg.2021.701689)
Supplement: Supplementary file 1 [file Table_1.DOCX]

Supplementary Material

# Factor Analyses

Regarding our target evaluation measures, we proposed four groups of variables in both of our studies: target likeability, prosociality, competence, and communal narcissism. An exploratory factor analysis (view Table S1) conducted in Study 1 reproduced this pattern in essence and revealed three factors: likeability, prosociality/competence, and communal narcissism. As both target prosociality and competence were purposely high due to our target description, and thus, variances were low, they did not emerge as two separate factors.

*Table S1. Exploratory Factor Analyses of the Target Evaluation Items in Experiment 1.*

| **Items** | **Factor** | | |
| --- | --- | --- | --- |
|  | **1** | **2** | **3** |
| Likeability item 1 | .89 |  |  |
| Likeability item 2 | .94 |  |  |
| Likeability item 3 | .94 |  |  |
| Prosociality item 1 |  | .63 |  |
| Prosociality item 2 |  | .61 |  |
| Prosociality item 3 |  | .74 |  |
| Prosociality item 4 |  | .73 |  |
| Prosociality item 5 |  | .46 |  |
| Prosociality item 6 |  | .54 |  |
| Ability item 1 |  | .68 |  |
| Ability item 2 |  | .86 |  |
| Ability item 3 |  | .86 |  |
| Communal narcissism item 1 |  |  | .85 |
| Communal narcissism item 2 |  |  | .97 |
| Communal narcissism item 3 |  |  | .54 |

*Notes. Factoring method: principal factor solution; rotation method: Oblimin; loadings smaller than .40 are not displayed.*
